# Topics of Public Interest

**Published:** 1909-10

**Authors:** 


### TOPICS OF PUBLIC INTEREST.

Four Thousand Consumptives Starve Yearly. Many Indigent Dying Cases are Being Sent to Southwest.

Cruel and inhuman practises are alleged in a statement given out a few days ago by the National Association for the Study and Prevention of Tuberculosis against the eastern doctors who persist in sending dying cases of consumption to the southwest.

Fully 7,180 persons hopelessly diseased with tuberculosis annually come to die in the states of California, Arizona, New Mexico, Texas and Colorado, most of them by order of their physicians. The statement, which is based upon the testimony of well known experts, and all available statistics, shows that at least 50 per cent. of those who go to the southwest every year for their health are so far advanced in their disease, that they cannot hope for a cure in any climate, under any circumstances. More than this, at least 60 per cent. of these advanced cases are so poor that they have not sufficient means to provide for the proper necessaries of life, which means that 4,315 consumptives are either starved to death, or forced to accept charitable relief every year.

It is not an uncommon thing, the National Association declares, for whole families, who can hardly eke out a living in the east, to migrate to the west in the hope of saving the life of some member of the family. In most instances, the abject poverty of such cases forces them to beg, or to live on a very low level. Often consumptives who cannot afford the proper traveling accommodations are found dead on the trains before reaching their destination. The resources of almost every charitable organisation in the southwest are drained every year to care for cases which would be self-supporting in their eastern homes.

It costs, on an average, at least $50 per month for the support of a consumptive in the southwest, including some medical attention. The National Association strongly urges no one to go to this section who has not sufficient funds to care for himself at least one year, in addition to what his family might require of him during this time. It is also urged that no persons who are far advanced with tuberculosis go to so distant a climate.

Consumption can be cured, or arrested in any section of the United States, and the percentage of cures in the east and the west is nearly the same. Any physician, therefore, who sends a person to the southwest without sufficient funds, or in an advanced or dying stage of the disease, is guilty of cruelty to his patient. Renewed efforts are being made to stop this practice, and to encourage the building of small local hospitals in every city and town of the country. Attempts are also being made in southern California and in Texas to exclude indigent consumptives or to send them back to the east.

### Eight Million Dollars Appropriated to Prevent Tuberculosis.—State Legislatures in Consumption Crusade.

Appropriations of over $4,000,000 for the suppression of consumption have been made by twenty-eight state legislatures in session during the past year, according to a statement issued recently by the National Association for the study and prevention of tuberculosis.

Since January 1, 1909, forty-three state and territorial legislatures have been in session. Of this number, twenty-eight have passed laws pertaining to tuberculosis; eight others have considered such legislation, and in only seven states no measures about consumption were presented. In all, one hundred and one laws relating to the prevention or treatment of human tuberculosis were considered and out of this number sixty-four were passed.

Of the sixty-four laws passed, fourteen were in reference to building new state institutions. New state sanatoria for tuberculosis will be built in Pennsylvania, Connecticut, where three will be erected, Arkansas, Oregon, South Dakota, North Dakota and Florida. In New York, North Carolina, Indiana, Massachusetts, New Hampshire and Maine, appropriations have been made for enlarging sanatoria, already being built or in operation. There are now twenty-seven states where such institutions have been established. Every state east of the Mississippi, except Illinois, West Virginia, Kentucky, Tennessee, South Carolina and Mississippi have provided hospitals for tuberculosis patients.

Five states, Illinois, New York, Ohio, Minnesota and Iowa, passed laws giving their county officers power to erect tuberculosis sanatoria without resorting to a special vote. In Maine, Connecticut, Rhode Island, New Jersey, Michigan, Iowa and Kansas, laws providing for the strict reporting and registration of tuberculosis were passed. Only five other states, including the District of Columbia, have such laws. The National Association considers laws of this character as the first requisite in an organised movement against tuberculosis.

Laws prohibiting promiscuous spitting in public places, were passed in Maine, Pennsylvania, New Jersey, Kansas and Connecticut. Spitters in these states will be prosecuted and fined.

Ten states have this year granted nearly $100,000 to be spent only for the education of the public about tuberculosis. In some states traveling exhibitions will be used, while in others lectures and literature will be the chief means of education. The states making provisions of this sort are California, New Jersey, Kansas, New York, Rhode Island, Iowa, Minnesota, Porto Rico, Delaware and Texas.

The statement of the National Association calls particular attention to one fact which shows the remarkable interest in anti-tuberculosis work, evoked during the past year, namely, that fully one-third of the $4,000,000 appropriated this year is by special legislation and for new work. The last Congress appropriated, in addition to this sum, nearly $1,000 000 for the maintenance of the three federal sanatoria in New Mexico and Colorado. It is estimated besides that the numerous county and municipal appropriations made or to be made for tuberculosis work for next year will aggregate at least $3,000,000, making the official public expenditures in the United States for the wiping out of tuberculosis at least $8,000,000.

---

DR. RAMON GUITERAS, OF NEW YORK, RETURNS FROM SOUTH AFRICA—SAW MR. ROOSEVELT IN THE JUNGLE. IN AN INTERESTING INTERVIEW, TELLS OF MEETING THE EX-PRESIDENT. DETAILS GIVEN OF THE REAL DANGER OF THE HUNT.

Professor Ramon Guiteras, of the Post-Graduate Hospital, classmate of Theodore Roosevelt and the first medical man to arrive here from East Africa since the ex-President entered the jungle, arrived home from Southampton, June 26, 1909, on the American liner Philadelphia.

Colonel Roosevelt did not know that his old friend was on his way out of the hunting fields, and was "delighted" on arriving at Mombasa to meet him.

"By George, Ramon," said the colonel, "I am glad to see you, and I would like much to have your weight." Mr. Roosevelt weighed more than 200 pounds when he said this, and Dr. Guiteras, who is over six feet tall, weighed under 180 pounds.

"I would gladly exchange with you, colonel," said the surgeon, "but I fancy you will be reduced in weight when you come out of the jungle."

Dr. Guiteras said the ex-President was in excellent health and condition when he parted company with him a day after the meeting in Mombasa, and declared that the hunting trip would be of great physical benefit to Mr. Roosevelt.

"The hunting is exceedingly interesting," said Dr. Guiteras, "but the exertion takes off flesh. I lost about twenty pounds, and I believe Mr. Roosevelt will take off about twenty-five pounds when he leaves Mombasa. I was so impressed with my experience in the jungle that I have already planned to return there at the first opportunity. I have not had a vacation in three years, and I took four months this season to hunt.

"There is nothing greatly to be feared from animals in Africa. The things to fear are unboiled water and insects. There is where the danger lies. I had a cook who tried to fool me when I demanded boiled water. to drink. I found he was not boiling the water for my canteens and I hired a special man to attend to that work alone. Before I came away from Africa, my cook died because he insisted on drinking the unboiled water. Then one must be careful of the 'tick,' or 'chigger,' which burrows into the skin under one's toe nails. If not plucked out at once an ulcer forms and the hunter is unable to walk.

"There is no way to avoid these insects by special footwear. They are, as a rule, prevalent at old camps, and the only way to avoid encountering them is to make camp in places that have never been used as such before. Infection from the spirillum is another thing to be avoided. It produces a fever, which if neglected causes an affliction of the eyes and facial paralysis."

Asked which he considered the most dangerous beast of the jungle, Dr. Guiteras replied promptly, "The wounded lion."

"The rhinoceros," he continued, "is an animal that needs careful watching, but he is more easily avoided than a wounded lion. The first sight one usually gets of a lion is his haunches. He has seen the hunter first and is stealing away. If you fire at him and wound him, he is at once the aggressor, and while you are stalking him he is stalking you, and is quick to attack. He will probably strike you to the earth with his paw, if he is successful, claw you and mawl you a few times and then bite you, but before he can accomplish much he is distracted and beats a retreat. Hardly more than twelve white hunters have been killed in Africa by lions.

"The rhinoceros is different in his methods. He has poor sight, but a marvelous scent, and will trail the hunter and charge him. There is only one way to avoid this beast and that is by running at him, when he charges. Assuming that the rhinoceros is in the pitcher's box and the hunter is at the bat, the way to avoid the animal is to run to first base, then to second, and while the rhinoceros is confused send a ball from an express rifle behind his shoulder."

"Dr. Guiteras said he had shipped to New York, twelve varieties of animals he had killed, all of which will be mounted here."

